# Assessment of Environmental Contamination and Environmental Decontamination Practices within an Ebola Holding Unit, Freetown, Sierra Leone

**DOI:** 10.1371/journal.pone.0145167

**Published:** 2015-12-21

**Authors:** Daniel Youkee, Colin S. Brown, Paul Lilburn, Nandini Shetty, Tim Brooks, Andrew Simpson, Neil Bentley, Marta Lado, Thaim B. Kamara, Naomi F. Walker, Oliver Johnson

**Affiliations:** 1 King’s Sierra Leone Partnership, King's Centre for Global Health, King's College London, and King’s Health Partners, London, United Kingdom; 2 The Hospital for Tropical Diseases, University College London Hospitals, London, United Kingdom; 3 Public Health England, Salisbury, United Kingdom; 4 Connaught Hospital, Freetown, Sierra Leone; 5 Department of Infectious Diseases and Immunity, Imperial College London, London, United Kingdom; 6 Clinical Infectious Diseases Research Initiative, Institute of Infectious Disease and Molecular Medicine, University of Cape Town, Cape Town, South Africa; Division of Clinical Research, UNITED STATES

## Abstract

Evidence to inform decontamination practices at Ebola holding units (EHUs) and treatment centres is lacking. We conducted an audit of decontamination procedures inside Connaught Hospital EHU in Freetown, Sierra Leone, by assessing environmental swab specimens for evidence of contamination with Ebola virus by RT-PCR. Swabs were collected following discharge of Ebola Virus Disease (EVD) patients before and after routine decontamination. Prior to decontamination, Ebola virus RNA was detected within a limited area at all bedside sites tested, but not at any sites distant to the bedside. Following decontamination, few areas contained detectable Ebola virus RNA. In areas beneath the bed there was evidence of transfer of Ebola virus material during cleaning. Retraining of cleaning staff reduced evidence of environmental contamination after decontamination. Current decontamination procedures appear to be effective in eradicating persistence of viral RNA. This study supports the use of viral swabs to assess Ebola viral contamination within the clinical setting. We recommend that regular refresher training of cleaning staff and audit of environmental contamination become standard practice at all Ebola care facilities during EVD outbreaks.

## Introduction

Ebola virus is transmitted through direct contact with blood and other bodily fluids of an infected person. Mechanisms of transmission through direct contact with infected patients and body fluids have been studied in some depth but environmental transmission of the virus, whilst acknowledged to be important, is incompletely understood [1][2][3]. As a result, there is limited evidence to guide environmental decontamination protocols.

Ebola Holding Units (EHUs) admit patients with suspected Ebola virus disease (EVD) for EVD diagnostic testing before transferring negative patients to the general hospital environment and positive patients to Ebola Treatment Centres (ETCs). There is therefore the risk of nosocomial transmission within EHUs and this has the potential to be high for three reasons. Firstly, Ebola virus has been shown to survive for a potentially significant period of time [4]; studies in laboratory environments under optimal conditions have recovered viable virus from solid surfaces from 8–26 days [5][6]. A recently published laboratory study using simulated West African conditions matched for relative humidity and temperature levels, recovered viable virus from surfaces after 5 days [7]. However, the different methodologies used in these studies from the matrices used to suspend the virus to differing recovery techniques, limit their applicability to the real world setting. Additionally, the clinical environment may be more hostile to the virus, due, for example, to ultraviolet light, which is known to deactivate the virus [8]. Secondly, studies have demonstrated that the infectious dose can be low (1–10 plaque forming units) [9] although these studies were based on animal models with largely artificial routes of administration of virus, such as intramuscular injection. Finally, EVD patients often have prominent “wet” symptoms (diarrhoea, vomiting or bleeding) that spread virus in the environment. The potential for nosocomial transmission is highest within EHUs that have a rapid turnover of suspect patients.

The only previous study conducted within an Ebola healthcare facility, found no evidence of environmental contamination at 31 swab sites. Ebola virus was only detected on the 2 visibly blood-stained positive controls [1]. The reliability of these results may have been reduced however due to environmental swab collection occurring after environmental decontamination, delays in sample processing and the interruption of the cold chain.

Epidemiological data supports the possibility of nosocomial transmission through indirect transmission via surfaces or fomites [10][11]. A study of risk factors for Ebola Virus Disease (EVD) in Uganda, after controlling for direct contact, found that sharing food or a sleeping place with a positive case were both independent risk factors for contracting EVD. One patient in the study was considered likely to have contracted the virus after using a blanket previously belonging to a positive case [2].

**Fig 1:**
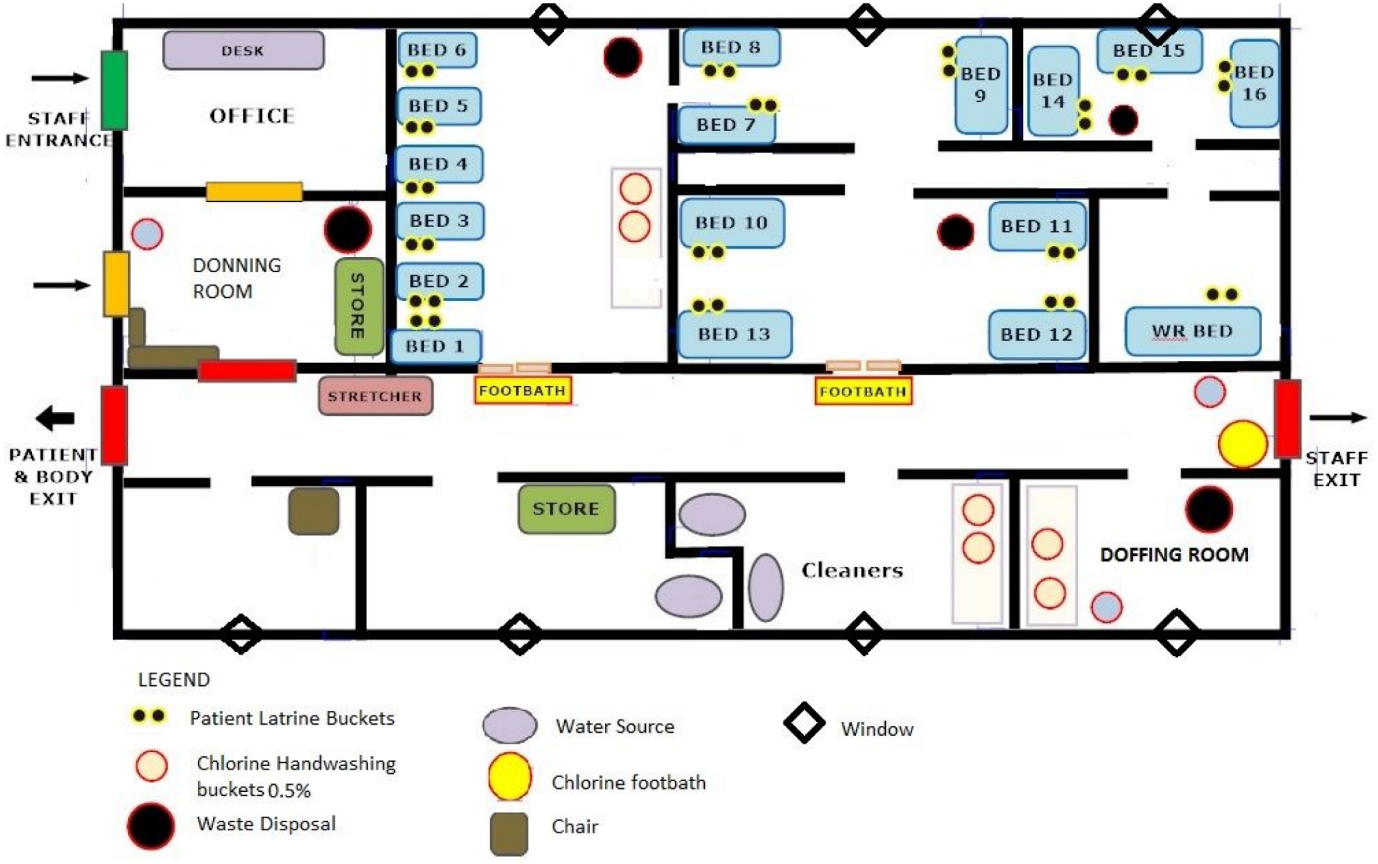
A schematic representation of Connaught Hospital EHU.

Our assessment was conducted at Connaught Ebola Holding Unit (EHU), a 16-bed facility situated within Connaught Hospital in Freetown, which is the principal adult medical tertiary referral hospital in Sierra Leone. The EHU is an adapted medical outpatient wing and was set up using pre-existing beds and structures (Fig 1); this significantly reduced the cost and lead-in time for opening the EHU compared to a purpose built facility. The EHU was established over two days in May 2014, soon after the first EVD cases were reported in the country, staffed by Sierra Leoneans who were supported by a small team of expatriate colleagues from King’s Sierra Leone Partnership. As of 1^st^ May 2015, Connaught Hospital EHU had seen 1400 suspect cases, of which 600 had tested EVD positive using RT-PCR; between May and December 2014 there was a median of 30 admissions per week, of which 64% cases were EVD-positive [12].

In this study we aimed to assess the effectiveness of our routine decontamination protocol in the EHU by swabbing environmental surfaces and testing for Ebola virus using RT-PCR. We hypothesised that Ebola virus would be detectable prior to decontamination on surfaces that had contact with an EVD patient or their body fluids, but that the virus would not be detectable after decontamination. The study was intended to improve our understanding of environmental contamination in order to reduce the risk of nosocomial transmission within the EHU.

## Method

The study was designed as an audit of routine decontamination practices at Connaught Hospital EHU. The unit had a total cleaning staff of 13, with 4 cleaners working in each of the three shifts. Cleaning staff were initially trained at Connaught Hospital according to the initial WHO Infection Prevention Control protocols [13] prior to undertaking work inside the unit. They received regular refresher training every four weeks throughout their employment and every two weeks following the rapid educational intervention (see Rapid Educational Intervention). The unit had five regular full cleans every 24 hours, which involved spraying or mopping all surfaces and floor, changing all bins and emptying patient latrines located by each bed. In addition, each bedside area was given a “deep clean” after a patient was discharged, consisting of changing all bed linen and latrine buckets and cleaning of mattress, bedframe, and all bedside surfaces. The cleaning fluid used was 0.5% sodium hypochlorite reconstituted in fresh batches six times a day from sodium hypochlorite powder.

The audit sampling procedures were undertaken immediately prior to, and then following, refresher training of cleaning staff. This allowed standard procedures and training to be evaluated. Cleaning staff were not informed of the audit to ensure routine cleaning standards were assessed. Three sets of swabs were collected and swab collection took place during a period of time when confirmed Ebola PCR positive patients were being managed in the EHU. Sample collection attempted to assess the “dirtiest environment” scenario by collecting samples in the early morning, before excessive ultraviolet light exposure and before the first daily routine cleaning. Initial sample collection (T0) took place immediately after discharge of EVD positive patients, the area surrounding the bed of the EVD positive patient was then given a “deep clean”, following the protocol described above. For each swab set, swabs were collected three times (corresponding to different time periods) at 15 pre-determined locations around the bedside of a RT-PCR positive patient, immediately after they left the facility. The three time periods were:

1. T0: Immediately after departure of an Ebola PCR positive patient, before environmental cleaning
2. T30: 30 minutes after environmental decontamination of bed and surrounding area
3. T60: 60 minutes after environmental decontamination of bed and surrounding area

Swab sets 1 and 2 were performed prior to the rapid educational intervention and Swab set 3 was performed after the intervention. In addition to the three sets of bedside swabs, an extended panel of swabs was taken both within the clinical area and outside of the EHU during set 3, using the same method. Swabs were collected by several of the authors (DY, CSB, PL, NW) dressed in full Personal Protective Equipment. ∑-Virocult^®^ swabs, combining a special foam head with a matching transport medium, were moistened in transport medium then rubbed over a 30cm by 30cm sampling area. Investigators performed full hand cleaning procedures in 0.5% chlorine solution between samples. Swabs were transferred to the Public Health England (PHE) laboratory at Kerrytown (transport time of 90 minutes) where samples were stored overnight at -20°C before undergoing RT-PCR. RT-PCR was carried out according to PHE standard operating procedures, including a no template control, negative extraction control and positive control, as validated in the UK and at three reference laboratories in Sierra Leone. The Trombley^™^ assay based upon the Ebola Zaire NP assay was used for all runs [14]. The positive threshold used was any CT value <40, the negative threshold was a CT value >40.

### Rapid Educational Intervention

After Bedside Swab Set 2, the environmental cleaning team were retrained. 10 members from various shifts (at least two from each shift) attending a 2-hour cleaning tutorial session. The tutorial involved practical demonstrations, accompanied by written materials in Krio and English which covered the correct use of 0.5% chlorine and correct floor and furniture cleaning techniques.

Following the tutorial, posters were placed around the EHU staff office detailing the correct environmental decontamination procedures. Refresher training courses were then scheduled every two weeks.

### Ethical considerations

This audit was performed with the approval of the medical superintendent of Connaught Hospital, Dr T.B. Kamara.

## Results

173 swabs were collected and processed following the protocol above. There were 16 positive samples in total. All positive samples were located around the immediate patient bedside (Table 1). Visible body fluid contamination was present on the mattresses at T0 in all three swab sets. There was also visible body fluid on the dirty glove at T0. No other environmental sample sites had visible body fluid contamination. There was no visible contamination present at T30 and T60 after the environmental deep clean at any sample site and therefore Ebola viral RNA detected at these times points was in the absence of visible contamination.

**Table 1:** RT-PCR results for swab sets 1, 2 and 3.

Location	Time (mins)	Swab Set 1	Swab Set 2	Swab Set 3
Floor—head of bed	0	NEGATIVE	NEGATIVE	NEGATIVE
Floor—head of bed	30	NA	NEGATIVE	NEGATIVE
Floor—head of bed	60	NEGATIVE	**POSITIVE CT 37.0**	NEGATIVE
Floor—middle of bed	0	NEGATIVE	**POSITIVE CT 34.6**	NEGATIVE
Floor—middle of bed	30	NA	**POSITIVE CT 32.4**	NEGATIVE
Floor—middle of bed	60	NEGATIVE	**POSITIVE CT 32.7**	NEGATIVE
Floor—foot of bed	0	NEGATIVE	NEGATIVE	NA
Floor—foot of bed	30	NA	NEGATIVE	NA
Floor—foot of bed	60	NEGATIVE	NEGATIVE	NA
Bedframe head of bed	0	NEGATIVE	NEGATIVE	NEGATIVE
Bedframe head of bed	30	NEGATIVE	NEGATIVE	NEGATIVE
Bedframe head of bed	60	NEGATIVE	NEGATIVE	NEGATIVE
Bedframe middle of bed	0	NEGATIVE	**POSITIVE CT 37.0**	NEGATIVE
Bedframe middle of bed	30	**POSITIVE CT 37.0**	**POSITIVE CT 35.1**	NEGATIVE
Bedframe middle of bed	60	NEGATIVE	**POSITIVE CT 39.3**	NA
Bedframe foot of bed	0	NEGATIVE	NEGATIVE	NEGATIVE
Bedframe foot of bed	30	NEGATIVE	NEGATIVE	NEGATIVE
Bedframe foot of bed	60	NEGATIVE	NEGATIVE	NEGATIVE
Mattress	0	**POSITIVE CT 31.2**	**POSITIVE CT 32.2**	**POSITIVE CT 37.4**
Mattress	30	NEGATIVE	NEGATIVE	NEGATIVE
Mattress	60	NEGATIVE	NEGATIVE	NEGATIVE
Latrine	0	**POSITIVE CT 36.2**	NEGATIVE	NEGATIVE
Latrine	30	NEGATIVE	NEGATIVE	NEGATIVE
Latrine	60	NEGATIVE	NEGATIVE	NEGATIVE
Wall	0	NEGATIVE	NEGATIVE	NEGATIVE
Wall	30	NEGATIVE	NEGATIVE	NEGATIVE
Wall	60	NEGATIVE	NEGATIVE	NEGATIVE
IV Pole	0	NEGATIVE	NEGATIVE	NEGATIVE
IV Pole	30	NEGATIVE	NEGATIVE	NEGATIVE
IV Pole	60	NEGATIVE	NEGATIVE	NEGATIVE
Bedside Table	0	NEGATIVE	NEGATIVE	**POSITIVE CT 39.2**
Bedside Table	30	NEGATIVE	NEGATIVE	NEGATIVE
Bedside Table	60	NEGATIVE	**POSITIVE CT 37.8**	NEGATIVE
Sharps Bin	0	NEGATIVE	NEGATIVE	NA
Sharps Bin	30	NA	NEGATIVE	NEGATIVE
Sharps Bin	60	NEGATIVE	NEGATIVE	NEGATIVE
Dirty Glove 1	0	NEGATIVE	**POSITIVE CT 34.9**	NEGATIVE
Dirty Glove 1	30	NEGATIVE	NEGATIVE	NEGATIVE
Dirty glove 2	0	**POSITIVE CT 36.9**	**POSITIVE CT 37.0**	NEGATIVE
Dirty Glove 2	30	NEGATIVE	NEGATIVE	NEGATIVE
Door Handle	0	NEGATIVE	NEGATIVE	NEGATIVE
Door Handle	30	NEGATIVE	NEGATIVE	NEGATIVE
Door Handle	60	NEGATIVE	NEGATIVE	NEGATIVE
Veronica Tap	0	NEGATIVE	NEGATIVE	NEGATIVE
Veronica Tap	30	NA	NEGATIVE	NEGATIVE
Veronica Tap	60	NEGATIVE	NEGATIVE	NEGATIVE
Apron	0	NEGATIVE	NA	NEGATIVE
Apron	30	NEGATIVE	NA	NEGATIVE

**Footnote**: Results are shown for samples collected at T0 (immediately after decontamination), T30 (30 mins after decontamination) and T60 (60 mins after decontamination). Cycle threshold (CT) values were quantified by the Public Health England (PHE) Trombley assay. NA = not applicable: swabs were either labelled ambiguously or sites were not sampled in that collection set.

The first bedside swab set demonstrates Ebola viral RNA in three locations: the patient latrine; the mattress; and the middle of the bedframe. The middle of the bedframe swabs detected virus at T30 but not at T0.

The second swab set demonstrates the most environmental contamination, with viral RNA detected in five locations around the bedside, the floor, the mattress, the bedframe and the bedside table. In addition, both gloves used by healthcare workers to help transport the patient to the ambulance had detectable virus prior to handwashing. Swabs of the bedframe and middle of the floor remained positive at T60 (i.e. after decontamination). The floor at the head of the bed and the bedside table gave positive results at T60 but not at T0.

The third data set was collected following the re-training of the isolation unit cleaning staff, as previously described. The only two positive results were from the mattress and at the patient’s bedside table at T0. These locations were subsequently found to be negative at T30 and T60 following environmental decontamination.

All of the extended panel of environmental swabs gave negative results (Table 2).

**Table 2:** RT-PCR results for Extended Panel of Swabs.

LOCATION	RT-PCR RESULT
Green zone—Desk	NEGATIVE
Green zone—document	NEGATIVE
Green zone—floor	NEGATIVE
Green zone—chlorine bucket	NEGATIVE
Green zone—door	NEGATIVE
PPE donning room—boot rack	NEGATIVE
PPE donning room—shelves	NEGATIVE
PPE donning room—Red Zone Boots side	NEGATIVE
PPE donning room—Staff boots underside	NEGATIVE
PPE room donning—Staff visor	NEGATIVE
Red zone—patient entrance	NEGATIVE
Red zone—morgue	NEGATIVE
Red zone—lab specimen box	NEGATIVE
Red zone Store room—patient clothes	NEGATIVE
Red zone patient store—floor	NEGATIVE
Red zone patient store—water	NEGATIVE
Red zone store room—medicine	NEGATIVE
Red zone store room—sharps bin	NEGATIVE
Red zone—chlorine sprayer	NEGATIVE
Red zone—stretcher	NEGATIVE
Red zone—wheelchair	NEGATIVE
Wheelchair post decontamination, after transfer of positive patient	NEGATIVE
Waste disposal container	NEGATIVE
Medicine tray	NEGATIVE
Environmental cleaning runoff liquid 1	NEGATIVE
Environmental Cleaning runoff liquid 2	NEGATIVE
Decontamination/Doffing room—floor	NEGATIVE
Decontamination room/doffing—Bin	NEGATIVE
Decontamination room/doffing—wall	NEGATIVE
Decontamination room/doffing—handwashing bucket	NEGATIVE
Decontamination room/doffing—handwashing bucket veronica tap 1/10	NEGATIVE
Decontamination room/doffing—chlorine footbath	NEGATIVE
Decontamination room/doffing—handwashing bucket 1/100	NEGATIVE
Decontamination/doffing room—handwashing bucket veronica tap 1/100	NEGATIVE
Incinerator area—wall	NEGATIVE
Incinerator area—door	NEGATIVE
Holding tent—benches	NEGATIVE
Holding tent—sides	NEGATIVE
Holding tent—floor	NEGATIVE
Screening tent—desk	NEGATIVE
Screening tent—documents	NEGATIVE
Screening tent—patient side rail	NEGATIVE
Boots 1 –pre decontamination	NEGATIVE
Boots 1 –post decontamination	NEGATIVE
Boots 2 –pre decontamination	NEGATIVE
Boots 2 –post decontamination	NEGATIVE
Boots 3 –pre decontamination	NEGATIVE
Boots 3 –post decontamination	NEGATIVE

## Discussion

The results demonstrate that Ebola virus RNA is detectable in limited areas around the patient bedside in the absence of visible contamination, in contrast to previous work in the clinical setting [1].

Swab set 1 detected a moderate amount of environmental contamination, followed by no environmental contamination at T60 and supports the hypothesis that cleaning was effective. One location was positive at T30, but not at T0; this may have been due to sampling a different area of the bedframe, displacement of the viral RNA from another location during the decontamination process, or from contamination from an adjacent patient’s bed space between sampling.

Swab set 2 results suggest the presence of heavy environmental contamination and an ineffective decontamination process, with the bedframe and middle of the floor remaining positive at T60 despite cleaning. Two other locations, the floor at the head of the bed and the bedside table, had detectable virus after environmental decontamination; again this may be due to sampling variability or displacement of viral RNA. It is possible that the contamination came from another patient, as there were many positive patients in the unit at this time and the area was not observed between swab sets. Incomplete decontamination could also be explained by a failure to effectively prepare this batch of the 0.5% sodium chloride cleaning solution. The greater amount of environmental contamination could be due to the patient being at a more advanced stage of the disease, with prominent “wet” gastrointestinal symptoms. This is also supported by the detectable viral RNA found on both gloves used by clinical staff transferring the patient.

Swab set 3 suggests a modest amount of environmental contamination followed by effective environmental decontamination. As we had expected, areas of contamination corresponded with areas of more direct and frequent contact by the patient, including the mattress, bedside table, and bedframe. The presence of viral RNA on the floor may be a consequence of direct contact by the patients or due to dispersion during urination, defecation or vomiting, as the latrine buckets are located on the floor next to the bedside. The uneven, rusted surface of the bedframes may have led to pooling of the virus and this may have further inhibited effective decontamination. All mattress specimens were positive at T0, highlighting the need for easily wipeable, non-absorbable mattresses. No viral RNA was detected on the walls, an area that could be contaminated by droplet spray directly from the patient. Indeed, apparent displacement of viral RNA from locations negative at T0 and positive at T30 or T60 appeared to occur in a downwards or lateral direction. The possible displacement of viral RNA to the floor in swab set 2 suggests the need for regular, effective decontamination of the floor and ideally individual soakaways for patient areas. It underlines the importance of chlorine foot baths for boot decontamination, demonstrated in Table 2, when moving between different areas within the EHU.

All of the extended panel of environmental swabs in Table 2 were negative. This provides reassurance that environmental contamination is largely limited to the immediate patient area. Importantly, no Ebola viral RNA was evident after routine hand-washing. Surfaces in the PPE doffing room were swabbed after staff that had cared for a positive patient had decontaminated, and these swabs were all found to be negative. Three sets of boots worn by clinical staff caring for the patient were negative. Samples from the wheelchair used to transfer the patient, also proved to be negative. All storage areas within the clinical area were negative. Run-off liquid from the environmental decontamination process as it left the patient area was also found to be negative.

Viral RNA was detectable 60 minutes after departure of the positive patient. Further operational research is needed to examine the time length of viral RNA detection in clinical settings. Current protocols suggest beds occupied by positive patients should remain empty for 24 hours post departure. During this outbreak there was huge pressure on the limited isolation bed capacity, particularly from October to December 2014, leading to faster than recommended bed turnover following departure of a positive patient. A greater evidence-base is needed to determine the safest time for a bed to be reoccupied, allowing for maximum bed capacity.

### Limitations

Swabs were collected from only a small sample area and the number of swab sets was small. We therefore cannot be certain that reduced viral RNA in swab set 3 was due to improved decontamination rather than sampling inconsistency. Further repetitions of the audit cycle would provide more generalizable results. All attempts were made to standardise the sampling technique including duration and exact location of sampling as detailed in the methodology, however technical variability in sample collection may be responsible for discordant results at T0, T30 and T60.

A CT threshold of <40 was selected to define a positive RT-PCR result. This is standard practice for clinical samples and therefore was deemed appropriate for this study. However, samples that tested negative may have contained viral RNA at a level below that detected by the assay. Whilst we hypothesise that sample sites that were negative at T0 and T30 but positive at T60 reflect transfer of contaminated material during cleaning, it is possible that this instead reflects a small difference in viral RNA concentration at a sampling site compatible with sampling variability, at the lower limit of detection of the assay, as detection of viral RNA was limited by sensitivity of the assay.

In addition, RT-PCR detection of viral nucleic acid does not confirm whether the virus is viable or not, as it may identify degraded virus, or virus inactivated by chlorine and non-viable as an infective agent. The very low nosocomial infection rate inside the Connaught EHU during this period suggests that actual environmental contamination that posed a risk of transmission must have also been very low [15]. All specimens collected have been archived, to enable extended PCR across multiple target sites combined with long range PCR or viral culture to be undertaken at a later date to assess viability of the virus. This is key to fully exploring the potential for nosocomial infection within EHUs.

## Conclusions

The incidence of nosocomial infection within Ebola healthcare facilities remains contentious. Health care facilities, with poor levels of infection prevention control have been implicated as important sites of amplification during filovirus outbreaks [16]. Studies from the current outbreak suggest low rates of nosocomial infection in new build, highly staffed facilities [17]. More recent publications show even lower positive readmission rates of between 1–3% across EHUs in Freetown, including Connaught Hospital EHU [15].

These results suggest that regular and effective environmental decontamination can reduce the presence of viral RNA around the bedside. It is not clear how the presence of viral RNA relates to transmission potential and further study of this is required, including the use of viral culture to ascertain whether PCR positive samples contain replication competent virus. However, our results suggest that if protocols are not closely adhered to, there is the potential for environmental contamination, which may increase the risk of nosocomial transmission. Environmental decontamination should be a priority in preventing nosocomial infection and thereby preventing healthcare facilities from acting as centres for amplification of the outbreak.

Our results support the use of purpose-designed swabs in viral transport media to assess surfaces and fomites for environmental contamination. The method offers a valuable tool to determine the effectiveness of decontamination protocols and the potential of nosocomial transmission via fomite transmission in the clinical setting. We recommend that regular audits of environmental contamination become standard practice at all Ebola care facilities during EVD outbreaks.
